# Identification of an Elusive *CBFA2T3::GLIS2* Fusion Variant in Acute Megakaryoblastic Leukemia by Whole Genome Sequencing

**DOI:** 10.1002/jha2.70254

**Published:** 2026-03-25

**Authors:** Yuna Lee, Jung Yoon Choi, Hyun Jin Park, Bo Kyung Kim, Hyun Kyung Kim, Yeseul Kim, Jung‐Eun Cheon, Sung‐Hye Park, Ji Hoon Phi, Ji Won Lee, Ryul Kim, June‐Young Koh, Hyoung Jin Kang

**Affiliations:** ^1^ Inocras Inc. San Diego California USA; ^2^ Department of Pediatrics Seoul National University College of Medicine Seoul National University Children's Hospital Seoul Republic of Korea; ^3^ Seoul National University Cancer Research Institute Seoul Republic of Korea; ^4^ Department of Laboratory Medicine Seoul National University Hospital Seoul National University College of Medicine Seoul Republic of Korea; ^5^ Department of Radiology Seoul National University Children's Hospital Seoul National University College of Medicine Seoul Republic of Korea; ^6^ Department of Pathology Seoul National University Hospital Seoul National University College of Medicine Seoul Republic of Korea; ^7^ Division of Pediatric Neurosurgery Seoul National University Children's Hospital Seoul National University College of Medicine Seoul Republic of Korea; ^8^ Department of Pediatrics Samsung Medical Center Sungkyunkwan University School of Medicine Seoul Republic of Korea

## Abstract

Advancements in genomic profiling have significantly improved the classification and treatment strategies for acute myeloid leukemia (AML). However, widely utilized molecular diagnostic techniques, including targeted gene panels, are often insufficient for detecting complex structural variants, cryptic fusions, and poorly characterized driver mutations. Here, we present the case of a 15‐month‐old female with pediatric acute megakaryoblastic leukemia (AMKL) who exhibited an atypical clinical presentation. Initial imaging revealed expansile lesions in the pelvic bones and vertebral bodies, prompting suspicion of malignancy. Conventional diagnostics, including immunohistochemistry and targeted sequencing, failed to identify a definitive oncogenic driver. Whole genome sequencing (WGS) identified a *CBFA2T3::GLIS2* fusion, leading to a revised AMKL diagnosis with a RAM immunophenotype. The patient underwent induction chemotherapy with cytarabine and mitoxantrone, followed by salvage therapy with venetoclax and azacitidine, resulting in morphologic remission. Subsequent haploidentical hematopoietic stem cell transplantation achieved remission, with ongoing hematologic recovery. This case underscores the limitations of conventional molecular assays in detecting cryptic fusions and highlights the critical role of comprehensive genomic profiling in refining subclassification and optimizing therapeutic strategies in pediatric AML.

1

The integration of genomic profiling into cancer diagnostics has become paramount, extending from prognostic predictions and elucidation of cancer origins to the identification of key driver genes for the selection of targeted therapies. [1] Historically, the French‐American‐British (FAB) classification, reliant on histological evaluation, was the primary method for acute myeloid leukemia (AML) classification. [2]. However, advancements in molecular diagnostics, particularly next‐generation sequencing (NGS), have facilitated the development of genomic classifications [3, 4]. This shift has been embodied by the adoption of the World Health Organization (WHO) classification, which incorporates genomic data to refine AML subclassification, thereby enhancing diagnostic precision and therapeutic decision‐making.

Pediatric acute megakaryoblastic leukemia (AMKL), also known as AML M7, accounts for approximately 10% of pediatric AML cases. In pediatric patients with non‐Down syndrome, this malignancy displays a particularly aggressive behavior and is associated with unfavorable outcomes. Within this subset, the most frequently identified chimeric oncogene is *CBFA2T3::GLIS2* fusion, which arises from a chromosome 16 inversion (inv(16) (p13.3q24.3)) [5]. Here, we present a case of AMKL with an extramedullary disease with normal peripheral blood counts characterized by a *CBFA2T3::GLIS2* fusion that was undetected by conventional diagnostic modalities and panel sequencing.

A 15‐month‐old girl presented to Seoul National University Hospital with a limping gait and left leg pain. Physical examination revealed limited hip range of motion due to pain. Neurological examination results were unremarkable. An initial complete blood count (CBC) test showed: white blood cell count of 12,020/µL, platelet count of 498,000/µL, and hemoglobin level of 12.6 g/dL. Peripheral blood smear examination showed normocytic normochromic red blood cells. White blood cells were normal in number, with 1% atypical lymphocytes. Simple radiography and magnetic resonance imaging (MRI) of the pelvis (Figure 1A) revealed expansile masses in the left ischium and lesions in the right pelvic bone as well as the L1 and L5 vertebral bodies. The lesions exhibited T2 intermediate and T1 low signal intensity with diffusion restriction and mild heterogeneous enhancement of the surrounding tissue. Consistently, 18F‐Fluorodeoxyglucose (18F‐FDG) positron emission tomography/computed tomography (PET/CT) demonstrated intense uptake in all lesions (Figure 1B). The patient underwent a core needle biopsy of the mass in the left ischium, and the pathological diagnosis indicated that the mass was consistent with an undifferentiated small round cell tumor with oval to round nuclei and inconspicuous nucleoli (Figure 1C,D). Immunohistochemical analysis revealed that the tumor cells were entirely negative for CD45, myeloperoxidase (MPO), and CD99 (Figure 1E)—markers typically positive in AML—as well as other markers such as CD20, CD3, and CD38, leaving no diagnostic clues. Targeted panel sequencing was conducted to confirm the diagnosis of small round cell malignancy. No actionable mutations were detected in targeted panel sequencing.

**FIGURE 1:**
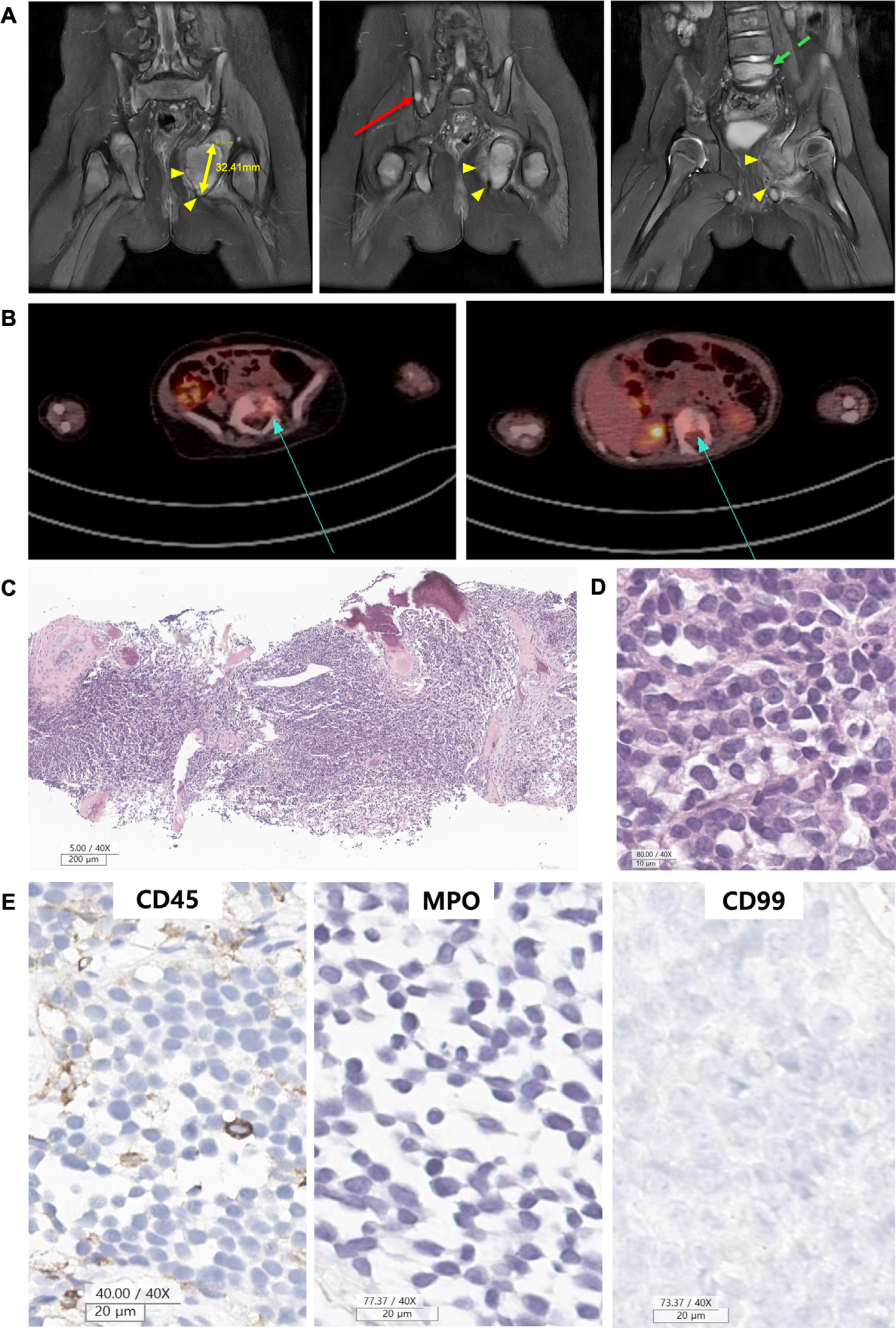
Findings from the conventional diagnostic approach prior to WGS. (A) Coronal fat‐suppressed T2‐weighted MRI showing a 32.41 mm lobulated contour mass involving the left ischium (yellow arrowheads), along with multifocal T2 hyperintense lesions in the right ilium (middle, red arrow), and L5 vertebral body (right, green dashed arrow). (B) 18F‐FDG PET/CT findings (L1 and L5 vertebral body). (C–E) Preoperative biopsy features from the spine (C1‐T1 level). (C, D) H& E staining results. (E) Immunohistochemical analysis results. CD45, MPO, and CD99. 18F‐FDG PET/CT; 18F‐fluorodeoxyglucose positron emission tomography/computed tomography, H& E; hematoxylin and eosin, MRI; magnetic resonance imaging, WGS; whole genome sequencing.

To facilitate a more precise molecular diagnostic approach, WGS was performed on specimens comprising blood and fresh frozen tumor tissue obtained through bone biopsy. Library construction and DNA extraction were performed in a CLIA‐certified laboratory. Genomic sequencing and analysis were performed using the WGS module of the CancerVision system (Inocras Inc., San Diego, CA, USA). Tumor tissues and normal blood samples were sequenced at depths of 40× and 20×, respectively. Tumor cell purity was 90%, and tumor mutational burden (TMB) was 1.05 mut/Mb, with 912 single‐nucleotide variants (SNVs), 2086 insertions/deletions (indels), and 2 structural variations (SVs). Mutational signature analysis based on single base substitutions (SBS) revealed 33.7% of SBS5 (clock‐like signature) and 30.4% of SBS3 (defective homologous recombination DNA damage repair). A closer examination of the SVs revealed a balanced inversion between *CBFA2T3* Intron 11 and *GLIS2* Intron 3. This inversion resulted in a fusion of *CBFA2T3* Exon 11 with *GLIS2* Exon 4 (Figure 2A), which was confirmed using RNA sequencing. Based on these results, AMKL was strongly suspected [6, 7], and bone marrow (BM) examination was performed. The BM aspiration revealed 21.8% blast cells (Figure 2B) with a specific surface marker expression profile, including CD45‐negative, CD56‐strongly positive, MPO‐positive, CD33‐positive, CD41‐positive, CD117‐positive, and CD34‐positive as determined by flow cytometry analysis (Figure 2C and Table S1). The blast cells demonstrated the RAM immunophenotype, characterized by strong CD56 expression and lack of CD45, along with megakaryocytic lineage markers (CD41 and CD117) and aberrant myeloid markers (MPO and CD33). Finally, the patient was diagnosed with AMKL with RAM immunophenotype [8]. A list of antibodies used for immunohistochemistry or flow cytometry (as applicable) is provided in Table S2. Further, targeted RNA sequencing with BM identified a *CBFA2T3*::*GLIS2* fusion transcript with a variant allele frequency (VAF) of 91.1% (Figure 2D). The patient subsequently underwent induction chemotherapy for AML with cytarabine and mitoxantrone. Follow‐up BM examination revealed persistent disease, with CD34‐positive blast cells comprising 7.0% of total nucleated cells in the BM aspirate and 60.5% of total nucleated cells in touch imprint. The patient did not achieve complete remission, despite several cycles of chemotherapy (high‐dose cytarabine and idarubicin/fludarabine/cytarabine). She achieved morphologic remission after receiving two cycles of venetoclax and azacitidine, but CD34 immunostaining remained persistently positive in focal areas. She underwent haploidentical hematopoietic stem cell transplantation (HSCT) with busulfan, fludarabine, and cyclophosphamide. One month after HSCT, BM examination showed remission with inCBC recovery. She is currently under continuous follow‐up.

**FIGURE 2:**
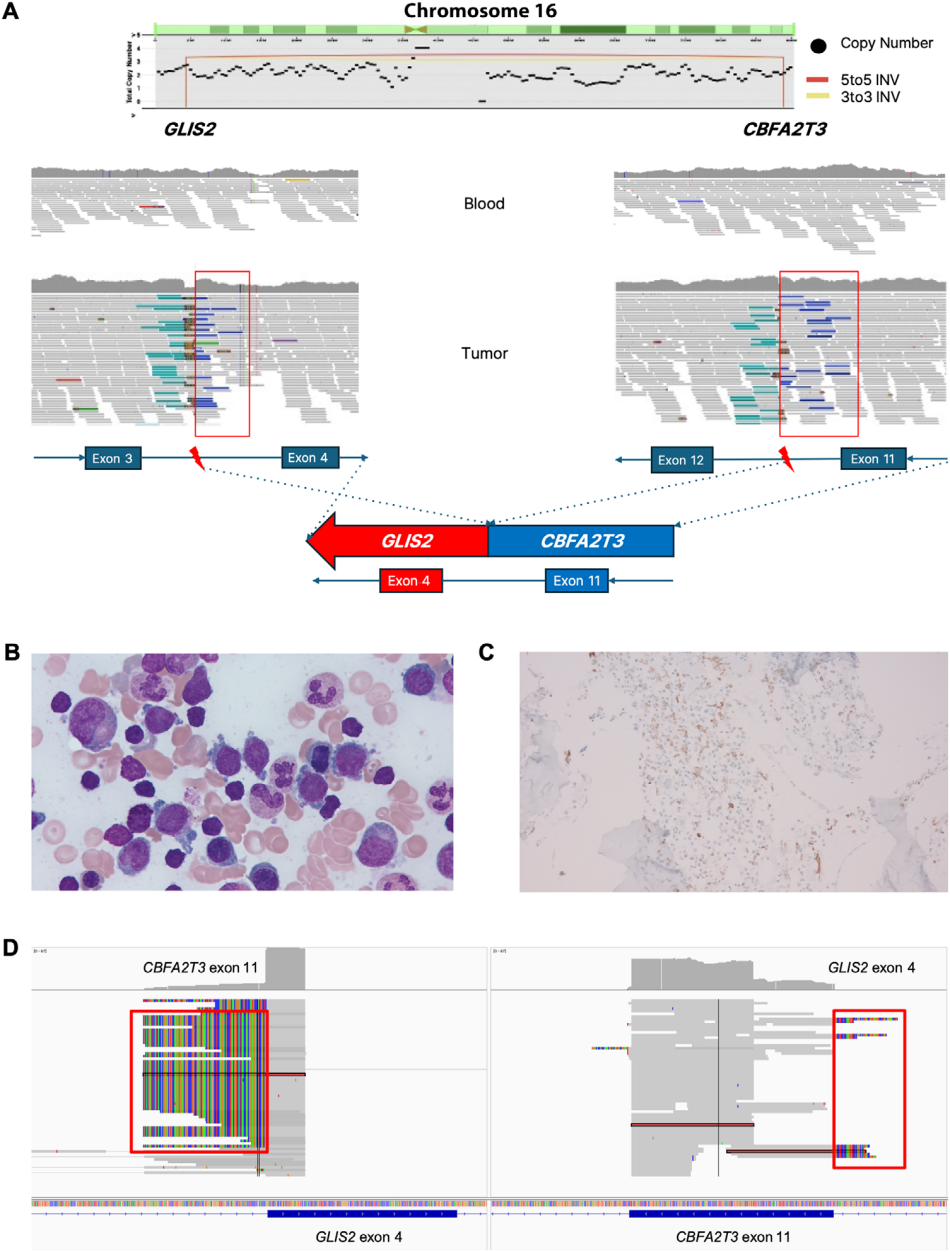
Identification of *CBFA2T3::GLIS2* fusion indicating AMKL through WGS (A) IGV (Broad Institute, Cambridge, MA) snapshot and schematic representation showing the *CBFA2T3* Exon 11 and *GLIS2* Exon 4 fusion caused by a 5′–5′ inversion on Chromosome 16. Navy‐colored reads aligned in opposite orientation indicate the inverted configuration of the genomic segment, and red boxes highlight clusters of paired‐end reads supporting the inversion breakpoint. The schematic diagram below illustrates the fusion junction between *CBFA2T3* and *GLIS2*. (B) H& E staining of BM aspiration showing blast cells. (C) Immunohistochemical staining of BM biopsy reveals increased CD34‐positive blast cells. (D) IGV snapshot of RNA sequencing reads demonstrating soft‐clipped reads within *CBFA2T3* and *GLIS2*; red boxes mark clusters where one portion of the read maps to *CBFA2T3* Exon 11 and the clipped segment aligns to *GLIS2* Exon 4, confirming the *CBFA2T3::GLIS2* fusion transcript at the RNA level. BM, bone marrow; H& E, hematoxylin and eosin; HSCT, hematopoietic stem cell transplantation; IGV, Integrative Genomics Viewer; WGS, whole genome sequencing.

This case presents AMKL characterized by a *CBFA2T3::GLIS2* fusion, presented by extramedullary lesions and localized pain. In typical cases of AML, abnormalities in the CBC or generalized symptoms lead directly to BM evaluation and timely diagnosis [9, 10]. In contrast, the present case manifested with extramedullary lesions resembling a small round cell solid tumor. An initial radiological examination revealed a bone‐involving mass, and CBC results remained within normal limits. These findings obscured the underlying hematologic nature of the disease and led to a suspicion of non‐hematologic pediatric solid tumors, prompting solid tumor‐focused evaluations, including targeted panel sequencing (Table S3), which ultimately delayed the diagnosis. Thus, the initial pathological evaluation based on morphological and immunohistochemical findings was inconclusive. Several reports have indicated that AML harboring the *CBFA2T3::GLIS2* fusion is more frequently associated with extramedullary involvement compared to other AML subtypes [11, 12]. In such cases, the presentation can closely mimic non‐hematopoietic solid tumors, often leading to substantial challenges to accurate and timely diagnosis. Although broad genomic approaches such as WGS are not routinely applied to all patients at initial evaluation, their consideration becomes essential in diagnostically challenging situations where standard clinical and histopathologic assessments fail to establish a definitive diagnosis.

The diagnostic challenges of AMKL due to similar atypical symptoms underscore the growing importance of molecular diagnostics in hematologic malignancies. Modern WHO classifications and clinical guidelines emphasize that molecular genetic analyses, such as fusion gene detection via WGS or transcriptomic sequencing, are essential for providing definitive diagnosis and establishing appropriate treatment strategies [13]. Nevertheless, in this case, the existing panel sequencing used in the hospital could not detect the *CBFA2T3::GLIS2* cryptic fusion. Although it could detect 151 RNA genes, *CBFA2T3* and *GLIS2* genes were not included in the panel because they were designed primarily for solid tumors.

Ultimately, WGS, an integrative diagnostic approach that encompasses the entire genomic structure, regardless of clinical symptoms, identified the *CBFA2T3::GLIS2* fusion gene, enabling a definitive diagnosis and allowing for appropriate treatment. Although she was refractory to multiple chemotherapy regimens, she achieved remission after HSCT, demonstrating the usefulness of HSCT in this disease. This case demonstrates the clinical utility of WGS for diagnosing challenging cancers. We identified this cryptic fusion using WGS, underscoring the diagnostic superiority of WGS in revealing critical genomic alterations that may elude other methods.

## Author Contributions

Conceived and designed the analysis: June‐Young Koh, Ji Hoon Phi, Ji Won Lee, and Hyoung Jin Kang. Collected the data: Jung Yoon Choi. Contributed data or analysis tools: Yuna Lee, Jung Yoon Choi, Hyun Jin Park, Bo Kyung Kim, Hyun Kyung Kim, Yeseul Kim, Jung‐Eun Cheon, Sung‐Hye Park, Ryul Kim, and June‐Young Koh. Performed the analysis: Yuna Lee, Hyun Jin Park, Bo Kyung Kim, Hyun Kyung Kim, Yeseul Kim, Jung‐Eun Cheon, Sung‐Hye Park, Ryul Kim, and June‐Young Koh. Wrote the paper: Yuna Lee, Jung Yoon Choi, Ryul Kim, and June‐Young Koh.

## Funding

This research was supported by a grant of the Korea Health Technology R&D Project through the Korea Health Industry Development Institute (KHIDI), funded by the Ministry of Health & Welfare, Republic of Korea (grant number: RS‐2024‐00440385 to June‐Young Koh).

## Ethics Statement

This study was approved by the Institutional Review Board of Seoul National University Hospital (IRB No. H‐2212‐155‐1391).

## Consent

Written informed consent was obtained from the patient(s) or their legal guardians for participation in this study and publication of relevant clinical data.

## Conflicts of Interest

The authors declare no conflicts of interest.

## Supporting information




**Supporting Table 1**: Flow cytometry analysis of BM biopsy revealing increased CD34‐positive blast cells*.
**Supporting Table 2**: List of primary antibodies used in immunohistochemistry or flow cytometry.
**Supporting Table 3**: Target gene list for the pediatric solid tumor target panel test.

## Data Availability

The data supporting the findings of this study are available from the corresponding author upon reasonable request.
