# Supplementary material for: Identification of an Elusive CBFA2T3::GLIS2 Fusion Variant in Acute Megakaryoblastic Leukemia by Whole Genome Sequencing
Source: EJHaem. 2026 Mar 25;7(2):e70254. doi: 10.1002/jha2.70254 (PMC13058918; doi:10.1002/jha2.70254)
Supplement: Supplementary file 1 — Supporting Table 1: Flow cytometry analysis of BM biopsy revealing increased CD34‐positive blast cells*. Supporting Table 2: List of primary antibodies used in immunohistochemistry or flow cytometry. Supporting Table 3: Target gene list for the pediatric solid tumor target panel test. [file JHA2-7-e70254-s001.docx]

Supplementary Table1. Flow cytometry analysis of BM biopsy revealing increased CD34-positive blast cells*

| **Marker** | **% Positive Cells** | **MFI**** | **Marker** | **% Positive Cells** | **MFI**** |
| --- | --- | --- | --- | --- | --- |
| TdT (Nuclear TdT) | 0% | - | CD34 (Stem cell) | 79% | +++ |
| MPO (Myeloperoxidase) | 74% | + | CD56 (NK cell) | 88% | +++ |
| CD2 (T cell) | 2% | - | cyt CD79a (B cell) | 13% | - |
| CD5 (T cell) | 3% | - | cyt CD3 (T cell) | 1% | - |
| CD7 (T cell) | 2% | - | cyt IgM (Cyt IgM) | 3% | - |
| CD10 (Common ALL Ag) | 11% | - | cyt CD22 (B cell) | 2% | - |
| CD19 (B cell) | 7% | - | CD3 (T cell) | 2% | - |
| CD20 (B cell) | 3% | - | CD14 | 2% | - |
| CD13 (Myeloid) | 27% | + | CD11c | 4% | - |
| CD33 (Myeloid) | 93% | +++ | CD64 | 3% | - |
| CD41 (Platelet GPIIb) | 81% | ++ | CD61 | 85% | + |
| CD117 (Myeloid) | 96% | +++ |  | | |

* The analysis was performed on bone marrow aspirated mononuclear cells (BMMCs). Marker expression was assessed in blasts (~10% of BMMCs) gated by negative CD45 expression and low side scatter (SSC).

** MFI; Mean Fluorescence Intensity (+ dim, ++ moderate, +++ bright, - negative for gated cells)

Supplementary Table2. List of primary antibodies used in immunohistochemistry or flow cytometry

| **Antibody** | **Target** | **Full name** | **Clone** | **Cat. No** | **Vendor** |
| --- | --- | --- | --- | --- | --- |
| CD3 | CD3 | Anti-CD3 antibody | 2GV6 | 790-4341 | VENTANA |
| CD20 (L26) | CD20 | Anti-CD20 antibody (Clone L26) | L26 | M0755 | DAKO |
| CD33 | CD33 | Anti-CD33 antibody | PWS44 | PA0555 | NOVO |
| CD34 | CD34 | Anti-CD34 antibody | QBEnd-10 | M7165 | DAKO |
| CD38 | CD38 | Anti-CD38 antibody | SP149 | 118R-14 | Cell Marque |
| CD45 (LCA) | CD45 | Anti-CD45 (Leukocyte Common Antigen, LCA) | 2B11+PD7/26 | M0701 | DAKO |
| CD56 | CD56 | Anti-CD56 (NCAM) antibody | 123C3.D5 | 156M-86 (1 mL) | Cell Marque |
| CD99 | CD99 | Anti-CD99 antibody | H036-1.1 | NCL-CD99 | NOVO |
| CD117 (C-kit) | C-Kit | Anti-CD117 (C-kit) antibody | POLY | A4502 | DAKO |
| Myeloperoxidase | Myeloperoxidase | Anti-human Myeloperoxidase antibody (MPO) | POLY | A0398 | DAKO |

Supplementary Table3. Target gene list for the pediatric solid tumor target panel test

| **List of target DNAs** | | | | | | | |
| --- | --- | --- | --- | --- | --- | --- | --- |
| *ACVR1* | *ADAM29* | *ADGRB3* | *ADGRG4* | *AIP* | *AKAP6* | *AKT1* | *ALK* |
| *APC* | *ARID1A* | *ARID1B* | *ARID2* | *ATM* | *ATOH1* | *ATRX* | *BAP1* |
| *BARD1* | *BCL3* | *BCOR* | *BCORL1* | *BRAF* | *BRCA1* | *BRCA2* | *BRIP1* |
| *C11ORF95* | *CBL* | *CCND1* | *CCND2* | *CCND3* | *CD300C* | *CD79A* | *CDH1* |
| *CDK12* | *CDK4* | *CDK6* | *CDKN1A* | *CDKN1B* | *CDKN2A* | *CDKN2B* | *CDKN2C* |
| *CHEK1* | *CHEK2* | *CIC* | *CREBBP* | *CSNK2B* | *CTDNEP1* | *CTNNB1* | *CYSLTR2* |
| *DAXX* | *DDX3X* | *DGCR8* | *DICER1* | *DIDO1* | *DKK2* | *DPYD* | *DROSHA* |
| *EED* | *EGFR* | *EMX2* | *EOMES* | *EPHA7* | *ERBB2* | *ERCC2* | *ERG* |
| *ETV6* | *EWSR1* | *EYA1* | *EZH2* | *FAM175A* | *FANCA* | *FANCD2* | *FANCL* |
| *FAT1* | *FBXW7* | *FGF3* | *FGF4* | *FGF6* | *FGFR1* | *FGFR2* | *FGFR3* |
| *FGFR4* | *FLG* | *FUBP1* | *FUS* | *GABRA5* | *GAD1* | *GFI1* | *GFI1B* |
| *GLI1* | *GLI2* | *GNA11* | *GNAQ* | *GNAS* | *GPR101* | *GSE1* | *H2AFX* |
| *H3F3A* | *HHIP* | *HIST1H3B* | *HIST1H3C* | *HRAS* | *IDH1* | *IDH2* | *IMPG2* |
| *JAK1* | *JUN* | *KBTBD4* | *KCNA1* | *KDM5C* | *KDM6A* | *KHDRBS2* | *KIT* |
| *KLF4* | *KMT2C* | *KMT2D* | *KRAS* | *LYST* | *MAB21L2* | *MACF1* | *MAML2* |
| *MAP2K1* | *MAP2K2* | *MAPK1* | *MAPK3* | *MDM2* | *MDM4* | *MED12* | *MEN1* |
| *MET* | *MLH1* | *MLH3* | *MN1* | *MRE11A* | *MSH2* | *MSH3* | *MSH4* |
| *MSH5* | *MSH6* | *MTOR* | *MYB* | *MYBL1* | *MYC* | *MYCN* | *MYL1* |
| *NEGR1* | *NF1* | *NF2* | *NOTCH1* | *NOTCH2* | *NOTCH3* | *NPR3* | *NRAS* |
| *NRL* | *NTRK1* | *NTRK2* | *NTRK3* | *OTX2* | *PALB2* | *PDGFRA* | *PDGFRB* |
| *PDZD2* | *PIK3CA* | *PLCB4* | *PMS1* | *PMS2* | *POLD1* | *POLE* | *POLQ* |
| *PPM1D* | *PPP2R2A* | *PRDM6* | *PRKAR1A* | *PRKCA* | *PTCH1* | *PTCH2* | *PTEN* |
| *PTPN11* | *RAD51B* | *RAD51C* | *RAD51D* | *RAD54L* | *RB1* | *RBM24* | *RELA* |
| *RET* | *RGPD3* | *ROS1* | *SETD2* | *SFRP1* | *SHH* | *SMAD2* | *SMAD4* |
| *SMARCA2* | *SMARCA4* | *SMARCB1* | *SMARCE1* | *SMO* | *SSTR2* | *STAG2* | *STAT3* |
| *STAT6* | *STK11* | *SUFU* | *SUZ12* | *SYNCRIP* | *TBR1* | *TBXT* | *TCF4* |
| *TERT* | *TET2* | *TNC* | *TP53* | *TRAF7* | *TRPS1* | *TSC1* | *TSC2* |
| *UNC5D* | *USP48* | *USP8* | *VHL* | *WIF1* | *YAP1* | *ZIC1* | *ZMYM3* |

| **List of target RNAs** | | | | | | | |
| --- | --- | --- | --- | --- | --- | --- | --- |
| *ABL1* | *AKT3* | *ALK* | *AR* | *AXL* | *B2M* | *BCL10* | *BCL2* |
| *BCL6* | *BCOR* | *BCR* | *BIRC3* | *BRAF* | *C11ORF95* | *CAMTA1* | *CBFB* |
| *CCNB3* | *CCND1* | *CCND3* | *CD274* | *CD28* | *CDK6* | *CHIC2* | *CIC* |
| *CIITA* | *COL6A3* | *CREB3L1* | *CREB3L2* | *CRTC1* | *CRTC3* | *CSF1* | *CTLA4* |
| *DDIT3* | *DDX31* | *DEK* | *DUSP22* | *EGFR* | *EPC1* | *ERBB2* | *ERBB4* |
| *ERG* | *ESR1* | *ETV1* | *ETV4* | *ETV6* | *EWSR1* | *EZHIP* | *FGFR1* |
| *FGFR2* | *FGFR3* | *FLI1* | *FOSB* | *FOXO1* | *FOXR2* | *FUS* | *GATA2* |
| *GFI1* | *GFI1B* | *GLI1* | *GLI2* | *GREB1* | *HMGA1* | *HMGA2* | *HTN3* |
| *IRF4* | *JAK2* | *JAZF1* | *KMT2A* | *KMT2D* | *MALT1* | *MAML2* | *MECOM* |
| *MET* | *MKL1 (MRTFA)* | *MKL2 (MRTFB)* | *MLF1* | *MLLT10* | *MN1* | *MSANTD3* | *MYB* |
| *MYBL1* | *MYC* | *NCOA1* | *NCOA2* | *NCOA3* | *NFKB2* | *NOTCH1* | *NOTCH2* |
| *NOTCH3* | *NR4A3* | *NRG1* | *NTRK1* | *NTRK2* | *NTRK3* | *NUP214* | *NUTM1* |
| *P2RY8* | *PAX3* | *PAX7* | *PAX8* | *PCSK5* | *PDCD1LG2* | *PDGFB* | *PDGFRA* |
| *PDGFRB* | *PHF1* | *PIK3CA* | *PKD1* | *PLAG1* | *PML* | *PPARG* | *PRDM16* |
| *PRKACA* | *PRKD1* | *PRKD2* | *PRKD3* | *PVT1* | *RAF1* | *RARA* | *RBM15* |
| *RELA* | *RET* | *ROS1* | *RSPO2* | *RSPO3* | *SLC44A1* | *SS18* | *STAT6* |
| *STIL* | *SUZ12* | *SYK* | *TAF15* | *TBL1XR1* | *TCF3* | *TCL1A* | *TERT* |
| *TFE3* | *TFEB* | *TFG* | *TGFBR3* | *THADA* | *TMPRSS2* | *TP63* | *TTYH1* |
| *TYK2* | *USP6* | *VAV1* | *VGLL2* | *WHSC1 (NSD2)* | *YAP1* | *YWHAE* |  |
